# Supplementary material for: Magnetic steering continuum robot for transluminal procedures with programmable shape and functionalities
Source: Nat Commun. 2024 May 4;15:3759. doi: 10.1038/s41467-024-48058-x (PMC11069526; doi:10.1038/s41467-024-48058-x)
Supplement: Supplementary file 1 — Supplementary Information [file 41467_2024_48058_MOESM1_ESM.pdf]

Supplementary Materials for

Magnetic Steering Continuum Robot for Transluminal Procedures with Programmable Shape and Functionalities

Liyang Mao†[1], Peng Yang†[1], Chenyao Tian†[1], Xingjian Shen[1], Feihao Wang[1], Hao Zhang*[1], Xianghe Meng*[1], and Hui Xie*[1]

1. State Key Laboratory of Robotics and Systems, Harbin Institute of Technology, 2 Yikuang, Harbin 150001, China

E-mail: haoz@hit.edu.cn, mengxianghe@hit.edu.cn, xiehui@hit.edu.cn

The PDF file includes:

Supplementary Text

Table S1

Figs. S1 to S21

Supplementary Reference

Other Supplementary Material for this manuscript includes the following:

Supplementary Movie 1 to 14

*To whom correspondence should be addressed

†These authors contributed equally to this work.

Supplementary Note 1 Supplementary Text.

The detailed preparation processes

The complete manufacturing process is illustrated in Figure 2a. The molten LMPA was injected by syringe throughout the silicone tube where a heating circuit had been pre-arranged (Step I). The tube sides were sealed with adhesive (silicone) after LMPA solidification (Step II). Upon curing the adhesive, more molten LMPA was injected into the silicone tube to expand the tube under pressure. The pressure in the silicone tube was maintained until the LMPA cooled to room temperature (Step III). When the LMPA is remelted, the pressure on the silicone tube enables the PTC to heal itself. After removal of the syringe, Follower could be obtained by directly gluing silicone tubing on the axis of the PTC (Step IV). The Guider came by embedding a permanent magnet into the tip and sealing it with adhesive (Step V).

Hydrophilic coatings need to be grown on the surface of both Guider and Follower to substantially reduce the coefficient of friction that impedes relative motion. A device was built to coat hydrophilic layers on PTC surfaces (Fig. S21a). The PTC was first cleaned with oxygen plasma for surface hydrophilicity and then completely submerged in the PVP solution. Afterward, the linear module lifted the PTC out of the PVP solution at a speed of 6 mm/s and then rotated the PTC in such a way that it was uniformly exposed for 3 min to the UV lamp (365 nm, 300 mw/cm^2). The ambient humidity should be suitable for preparing the hydrogel layer. Otherwise, the mechanical robustness would be significantly reduced (Fig. S21b). We calculated the friction coefficient by using the friction tester (BioNational Biomaterials company) to measure the forces required to pull PTC with and without the hydrophilic layer at a constant speed (10 mm/s) under a normal force of 3 N in water. The measurements showed that hydrogel coating of approximately 20 μm thickness (Fig. S21c) significantly reduces the friction coefficient and exhibited sufficient mechanical robustness.

Calculation of the inclination angle of the Guider's tip induced by the magnetic field

The segment sticking out was modeled with an Euler–Bernoulli beam model assuming a constant curvature along the length of the segment. We assume that the plane sections of the Guider remain in-plane and

perpendicular to the neutral-fiber axis after deformation, and the shear deformation is negligible, in which case, we have

$$k = \frac{T_{\mathrm{m}}}{E_{\mathrm{G}}^{\mathrm{f}} I_{\mathrm{G}}} \tag{S1}$$

where T_{m} is the magnetic torque, $E_{\mathrm{G}}^{\mathrm{f}}$ is the equivalent Young's modulus of the flexible Guider, I_{G} is the area moment of inertia of Guider, and k is the segment constant curvature. Under an external uniform magnetic flux density, denoted by a vector **B,** the tiny permanent magnet embedded in Guider's tip, haracterized by a magnetic moment **m**, experiences a magnetic torque. The torque norm can be expressed as

$$T_{\mathrm{m}} = Bm\sin(\gamma - \theta) \tag{S2}$$

where B and m are the norms of the magnetic flux density and the magnetic moment, respectively, γ represents the magnetic field inclination angle, and θ denotes the inclination angle of the tip. From Eq. S2, we notice that a misalignment angle γ-θ between the external magnetic field and the permanent magnet direction affects the torque applied to the tip. The conversion between tip inclination angle and curvature θ=kl is brought into the combination of Eq. S1 and S2, we can reach the following analytical expression for the inclination angle of the Guider's tip with length l

$$\theta = \frac{lBm}{E_{\mathrm{G}}^{\mathrm{f}} I_{\mathrm{G}}} \sin(\gamma - \theta) \tag{S3}$$

Ultrasound-based reconstruction of phantom vasculature and localization of the robot therein

First, to plan the path of the robot, a digital model of the phantom vasculature needs to be reconstructed. The ultrasound probe was fixed on a 4-DOF platform, capable of moving in 3D and rotating axially. Limited by the length of the ultrasound probe, the probe needed to scan the phantom vasculature multiple times in parallel (Fig. S7). Combining the image data captured by the US (Fig. S7 i-v) and the position data recorded by the 4-DOF platform, a point cloud can be obtained and a digital model of the phantom vasculature can be generated. Based on the digital model, the operator planned a path consisting of multiple arc segments to connect the start and end points. The control parameters for each segment were then calculated based on the kinematic model. The ultrasound probe is perpendicular to and scans along the planned path. Before

deployment, the ultrasound probe scanned once along the path to capture the background image. Then the ultrasound probe scanned once along the path during each motion cycle. The captured image was subtracted from the background image to obtain the robot's position with respect to the ultrasound probe, which in combination with the absolute coordinates of the ultrasound probe was used to determine the overall position and shape of the robot and represent it in the digital model.

Drug delivery to the gastric lesion

The porcine upper gastrointestinal tract was chosen as the experimental setting, including the esophagus, stomach, and duodenum. Mucus was removed several times with Pronase Granules to prevent visual field impairment due to minicamera contamination. A miniature camera was mounted in the stomach to record the complete movement of the robot. Two red markers were sutured to the lower part of the stomach to act as a lesion area. Gas was continuously pumped through the esophagus, and the duodenal junction was sutured to keep the entire stomach inflated. The robot was integrated with multiple functional modules, including a vision module, a drug delivery module, and a cleaning module to accomplish targeted drug delivery. The vision module can observe the environment and locate lesion areas. The drug delivery module can spray the drug to the target area. The cleaning module can spray water in a radial direction to remove mucus and air bubbles from the front of the minicamera.

Supplementary Note 2 Supplementary Tables

Table S1 Performances of existing variable stiffness continuum robots in terms of external diameter, Max. elasticity modulus, modulus ratio, and response time

Induction	Strategies	External diameter (mm)	Max. stiffness	Stiffness ratio	Response time (s)
Structural	Jamming-based stiffening	32[2]	~2.5MPa[2]	~10^2MPa[3]	Millisecond[3]
	Locking of relative motion[1]	12	loads of 1-5N	-	Millisecond
Material	Shape Memory Polymer	1.3[4]	~3GPa[4]	~90[4]	7[5]
	Low–melting point alloy	1[6]	1.4GPa[7]	45[7]	2-10[8]

The values correspond to the best results reported in the literature and may come as a result from different papers.

Supplementary Note 3 Supplementary Figures

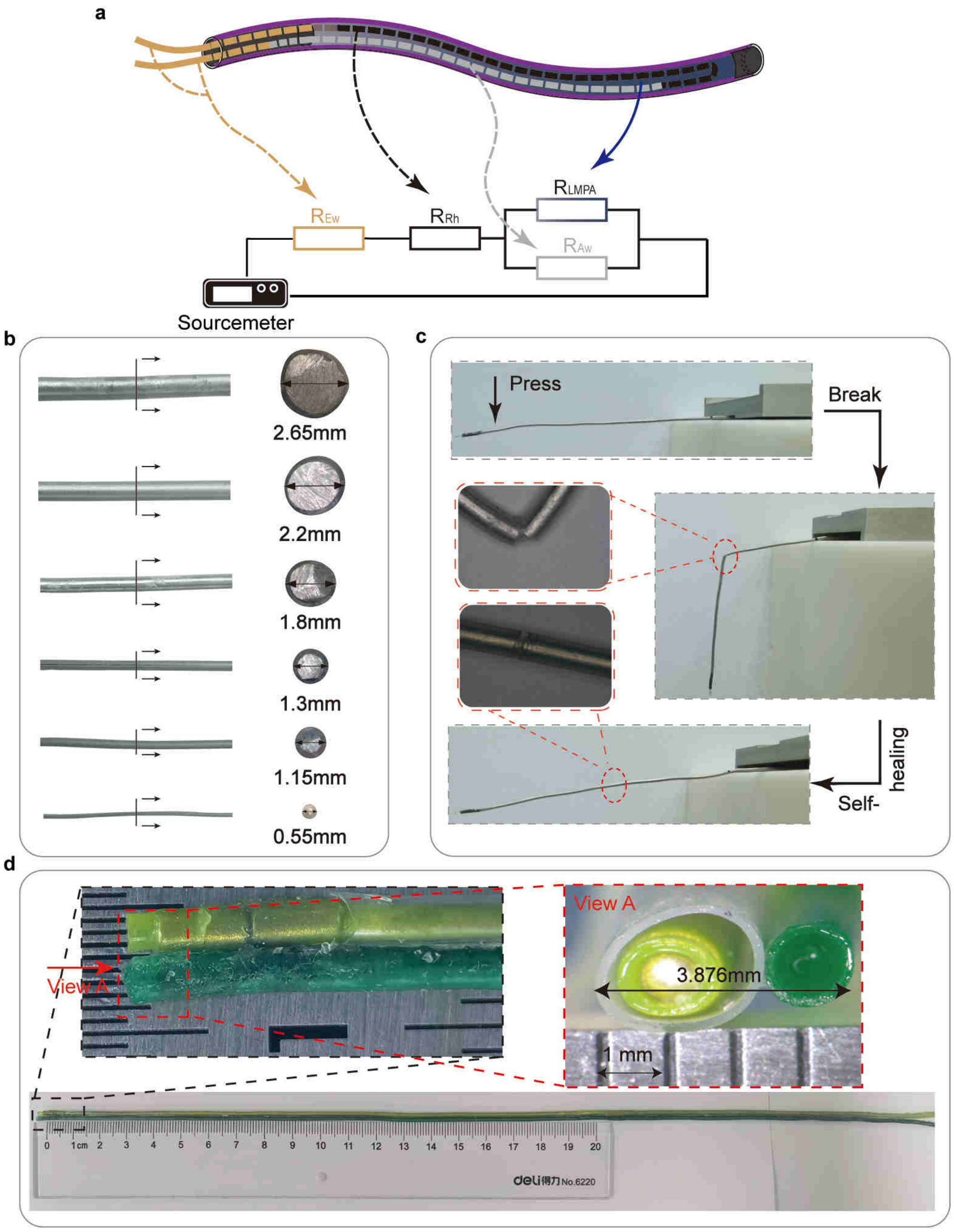

Figure S1. Construction and self-healing of phase transition component (PTC). (a) Internal heating circuit of PTC. Energy is mainly consumed by the resistive heater. The temperature is monitored indirectly by the detection of LMPA resistance. When LMPA breaks, the silver wire preserves the heating circuit intact. **(b)** End view of PTC in various sizes. PTC size can be further reduced if the mission is confined to a surface where gravity does not disturb the shape. **(c)** Self-healing property of PTC. Due to the continuous pressure exerted by the silicone tube on the LMPA, the fractured PTC can overcome the LMPA's interface phenomena at the fracture surface after melting, thereby achieving self-healing. **(d)** Overall, partial and sectional views of the robot.

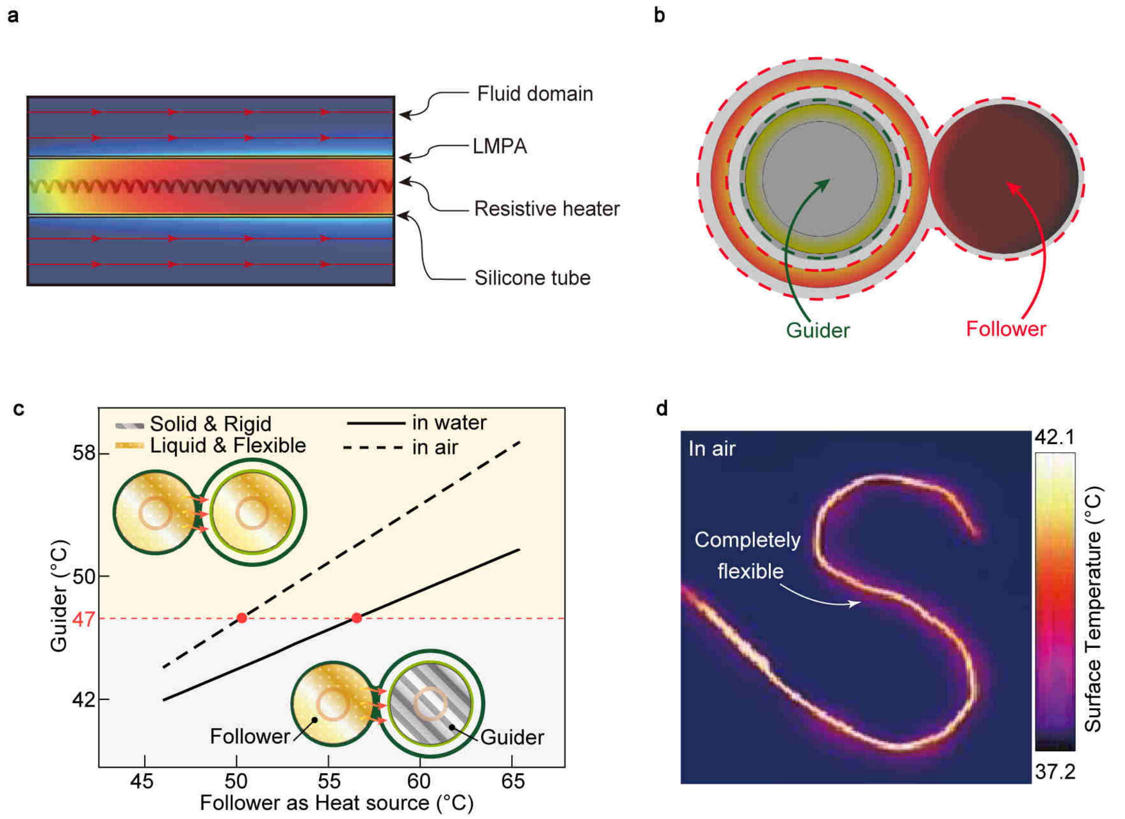

Figure S2. Temperature management of PTC. (a) Simulation of the minimum heating current in the fluid environment. **(b)** Simulation of the influence of the stable temperatures of separately heated Guider or Follower on each other. (**c**) The influence of the heated Follower's stable temperature on the Guider was obtained by numerical simulation. (**d**) PTC's surface temperature is taken by a thermal imager. During operation. The surface temperature of PTC can stabilize at 42°C in 37°C air environment (in a constant temperature oven).

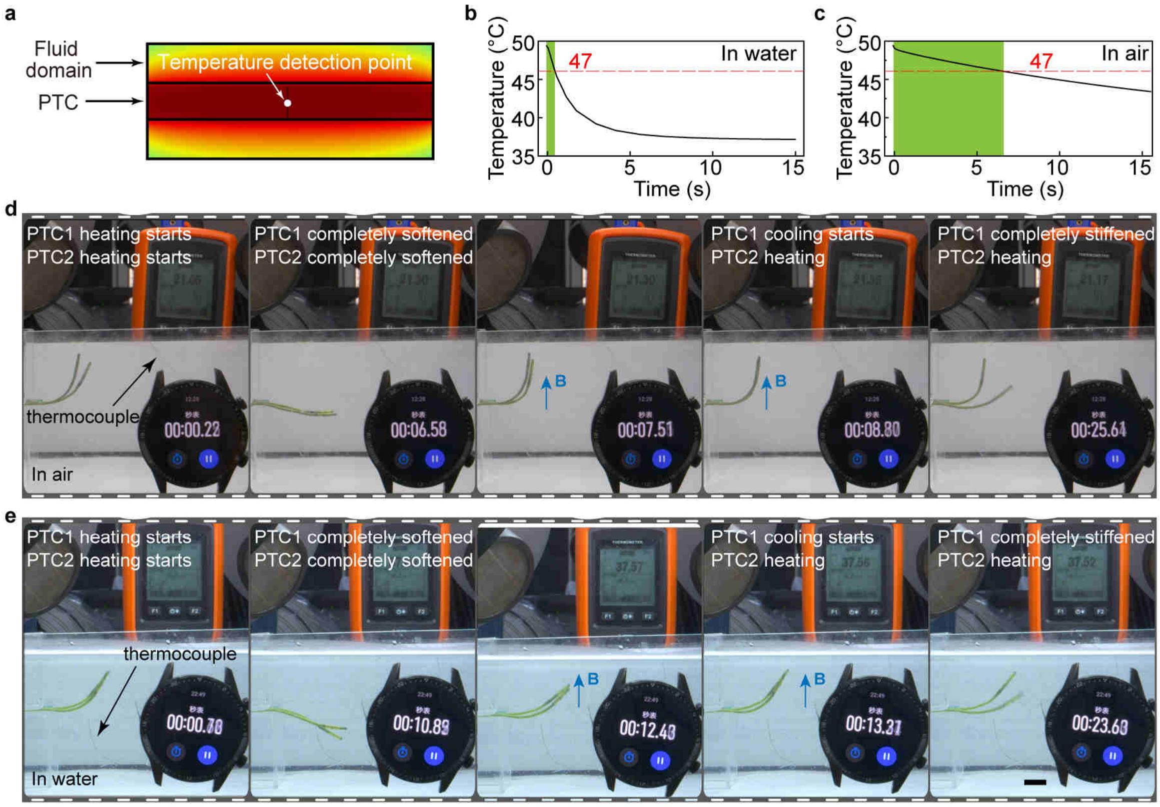

Figure S3. Heating and cooling of PTC. (a) Cooling Simulation of PTC. **(b)** Simulation results of PTC's cooling time in water. (**c**) Simulation results of PTC's cooling time in water. (**d**) and (**e**) First, both PTCs are heated simultaneously, and after about 6s (in air) and 10s (in water) they become completely flexible and bend downward under the force of gravity. Then, both PTCs were bent upward in response to the applied magnetic field. At the same time the heating of one PTC stops and it cools naturally in the environment. After about 16s (in air) and 10s (in water), the magnetic field was withdrawn and the heated PTC sagged under gravity, while the cooled PTC kept its shape unchanged, which was seen to become completely rigid. Scale bars, 10 mm.

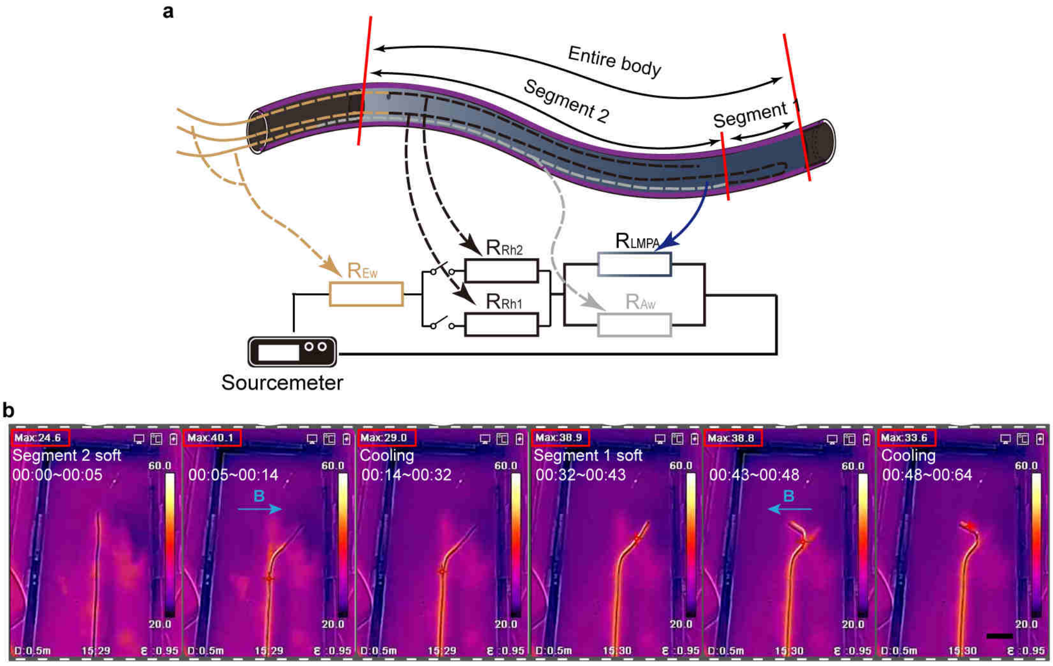

Figure S4. PTC with segmented variable stiffness capability. (**a**) Internal heating circuit of PTC. An additional resistive heater is added. (**b**) First, the resistance heater 2 was heated to soften segment 2. Then the PTC was bent and cooled under magnetic torque. Next, a suitable current was passed to the heating resistive heater 1 so that segment 1 was flexible while segment 2 remained rigid. Finally, a magnetic field was applied to bend the tip of the PTC. Scale bars, 10 mm.

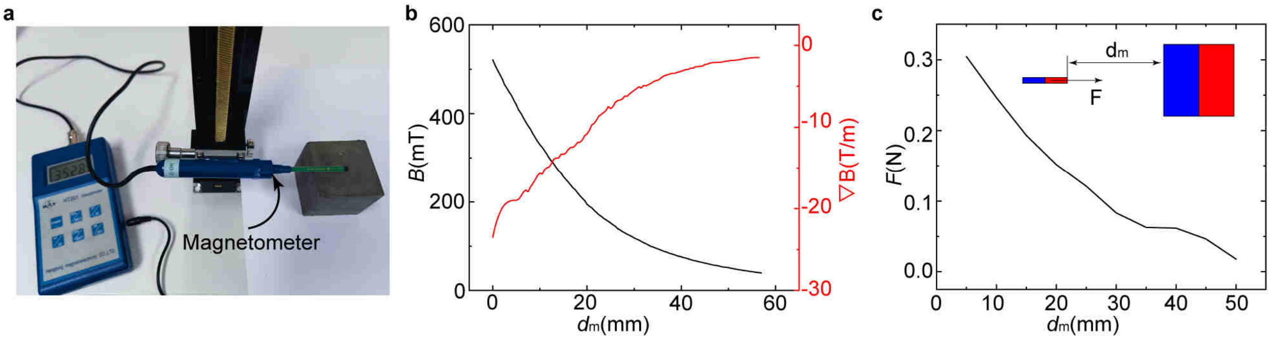

Figure S5. Gradient force generated by the permanent magnet on the tip. (**a**) A magnetometer is mounted on a displacement stage to measure the magnetic field around a permanent magnet. (**b**) Magnetic flux density and magnetic flux density gradient versus distance from the surface of the permanent magnet (50×50×50 mm) at the neutral axis. (**c**) Simulation results of the pull force on the magnet at the tip of the robot as a function of distance.

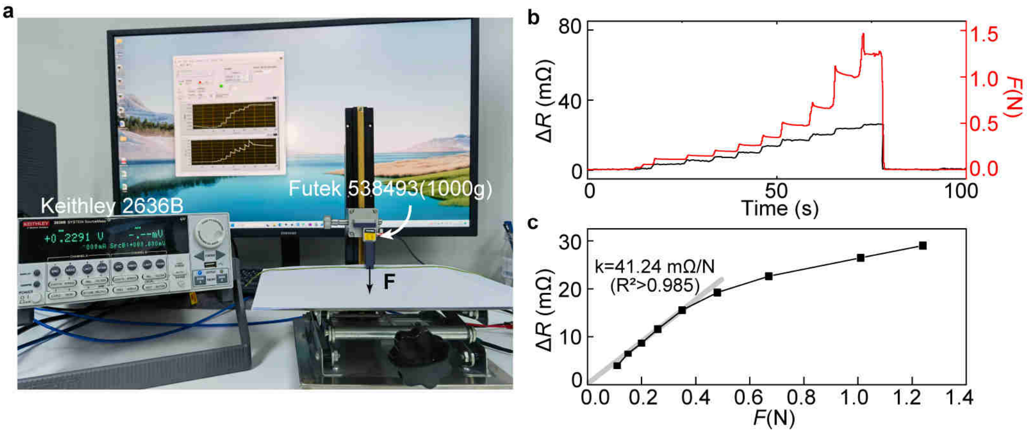

Figure S6. Characterization of the sensitivity of the knot to radial forces. (**a**) Measurement system. The system consisted of two mechanical stages, a force gauge (Futek 538493) for gradually ramping up force with precise control, and a sourcemeter (Keithley 2636B) for recording real-time resistive responses. (**b**) Real-time recording of the amount of resistance change and force. (**c**)Relationship between the amount of resistance change and radial force.

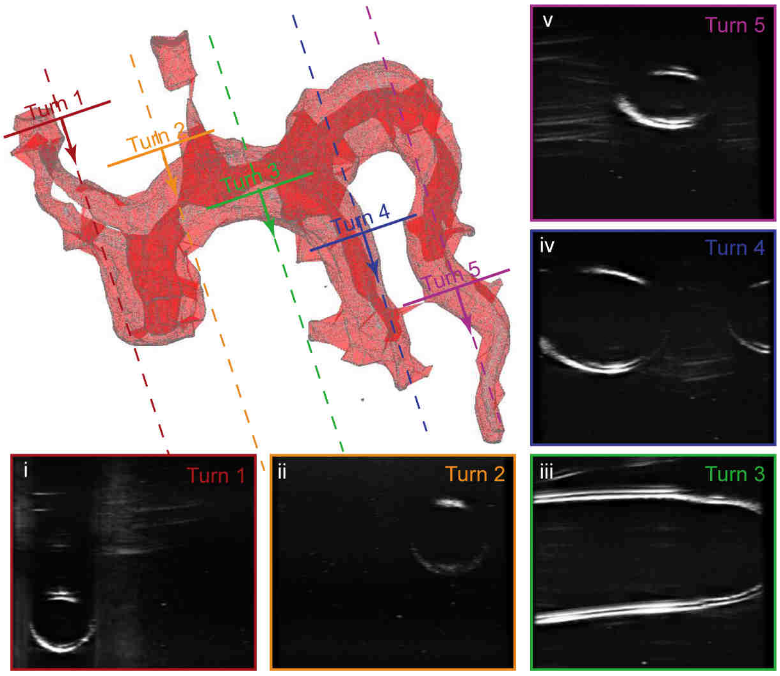

Figure S7. Vascular network model reconstruction. The ultrasound probe, clamped by a 4-DOF platform, scans the vascular network in parallel and acquires ultrasound images **i-v**. By combining probe position tracking and ultrasound image processing, the digital model of the vascular network can be reconstructed.

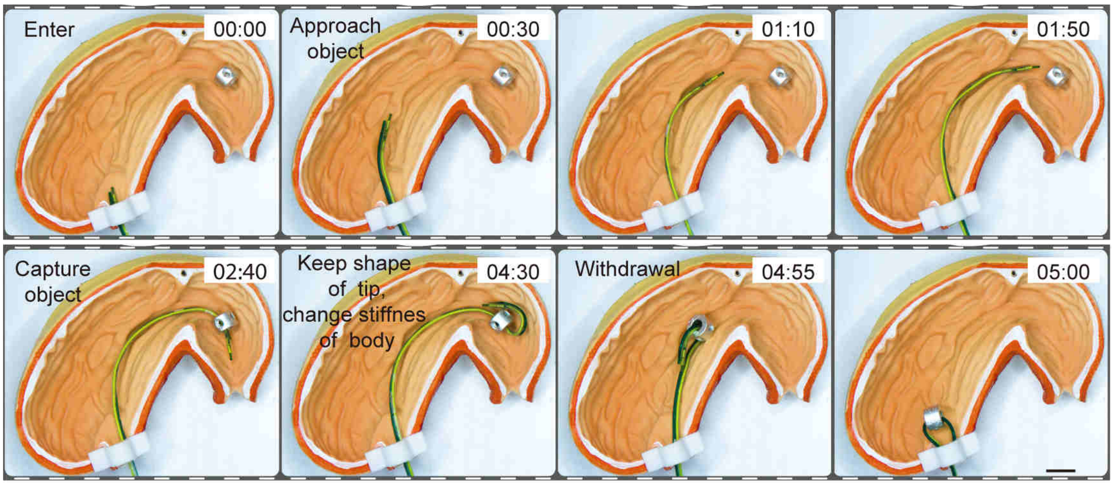

Figure S8. Removal of foreign bodies from the stomach. The robot can enter the stomach to hook the foreign body and then maintain the stiffness of the distal end while withdrawing. Scale bars, 20 mm.

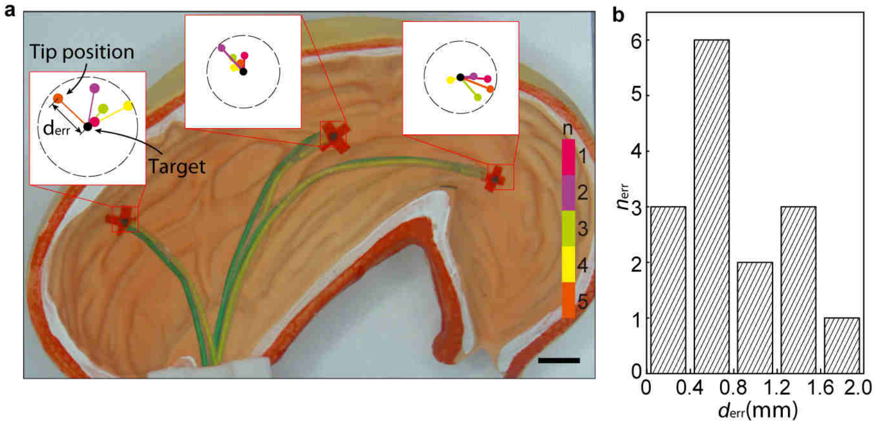

Figure S9. Precision and accuracy testing of the continuum robot. (**a**) Localization accuracy of a continuum robot reaching the same position multiple times (n = 5) in a row. The error of the robot's tip to the target is represented in the embedded figure. Scale bars, 10 mm. (**b**) Distribution of localization errors of the continuum robot.

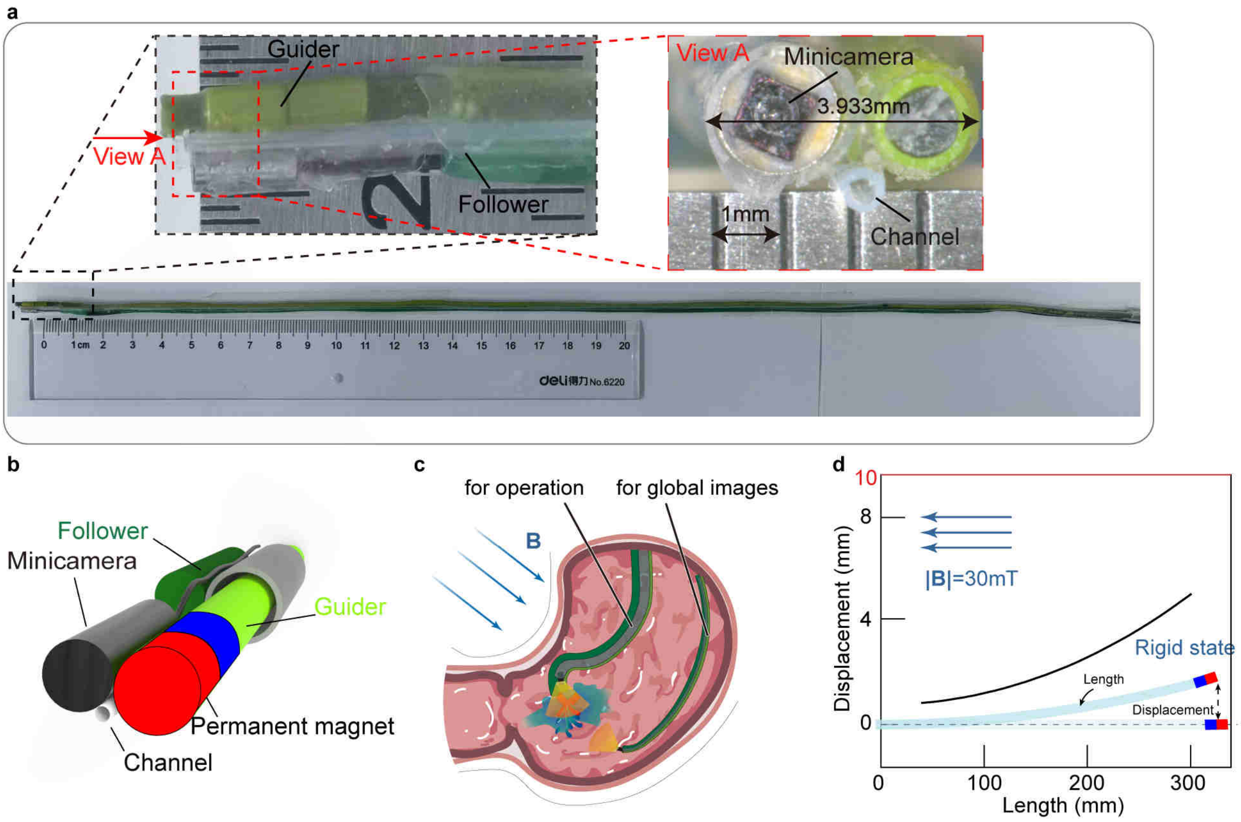

Figure S10. Robot equipped with endoscopic tools. (a) Overall, partial and sectional views of the robot with supplementary tools (a working channel and a minicamera with built-in illumination). **(b)** The supplementary tools are loaded in the Guider's tip, which keeps the overall size from increasing. **(c)** In the clinic, another continuum robot can be used to arrange the minicamera for global images. **(d)** Relationship between protrusion length of the guider and its tip displacement under a magnetic field. The confined space inside the body limits the length of the deployed robot, so the magnetic field has little effect on the rigid robot

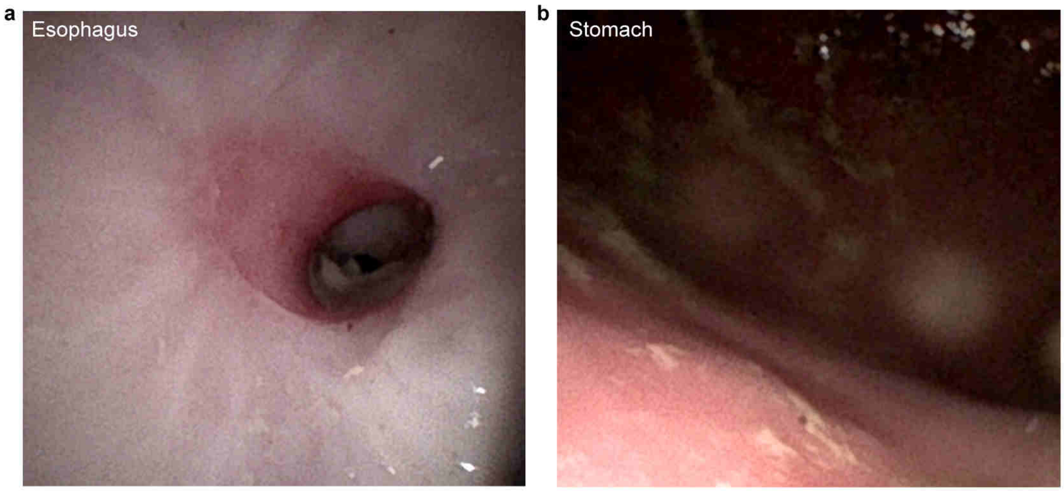

Figure S11. Commercial Gastroscopy. Following in vivo experiments, the upper gastrointestinal tract of the live porcine model was examined by a commercial gastroscope to evaluate the damage.

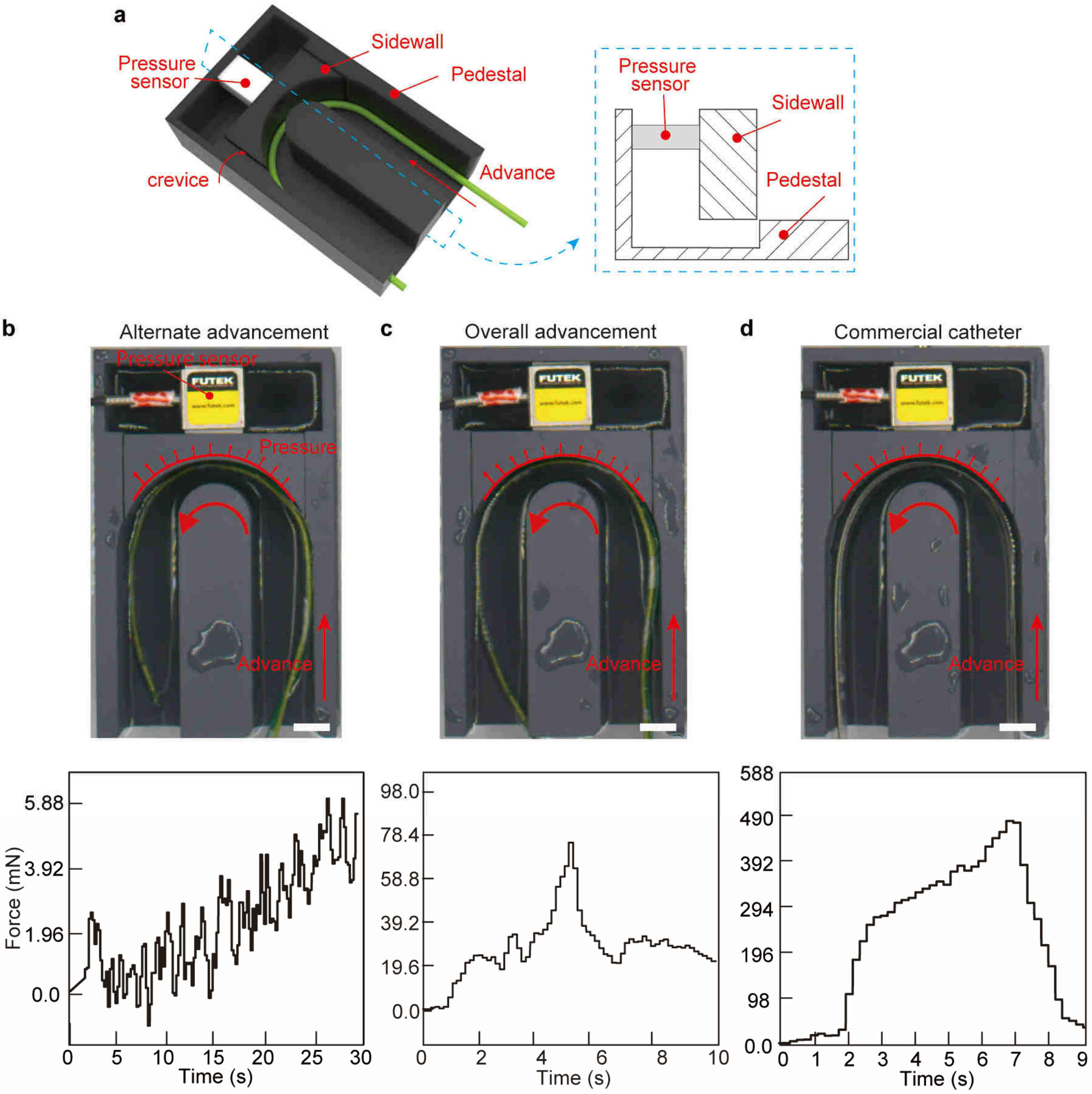

Figure S12. Force on the sidewall during a 180° turn. (**a**) Measuring the force on the sidewalls of a continuum robot as it moves forward. One side of the pressure sensor is fixed on the pedestal and the other side is connected to the corner sidewall. To avoid the effect of friction, the corner sidewall is not in contact with the pedestal. The force of the robot on the sidewall is captured by the sensor. (**b**) Pressure on the corner wall when two PTCs are alternately advanced. (**c**) Pressure on the corner wall when both PTCs are softened and advanced at the same time. (**d**) Pressure on corner wall when commercial catheter (OD=15Fr) is advanced. Scale bars, 10 mm.

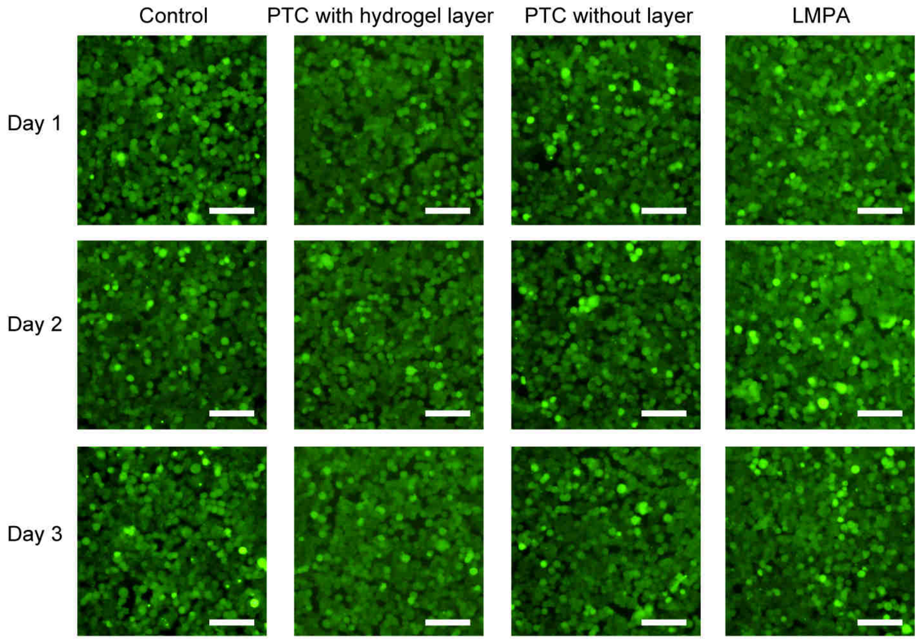

Figure S13. Cytotoxicity Test of PTC and LMPA on HUVEC Cells. PTC with hydrogel layers, PTC without layers, and LMPA were placed in the culture environment of HUVEC cells for 3 days for cytotoxicity test. Scale bars, 100 μm.

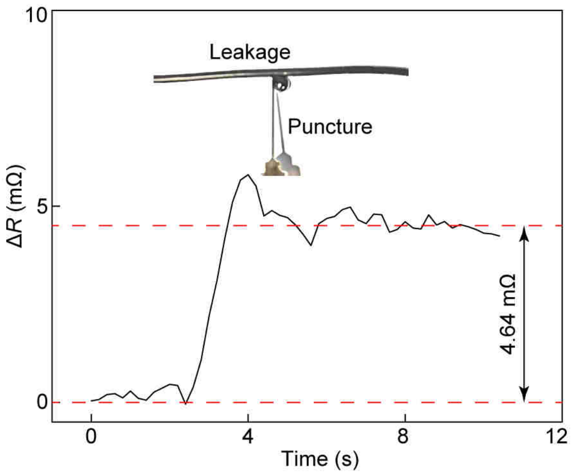

Figure S14. Leakage detection in PTC. If the PTC is punctured, the pressure inside it will squeeze out the LMPA, causing the radial dimension to decrease and the resistance to rise. If an abnormal rise in resistance is detected, the continuum robot should be withdrawn quickly.

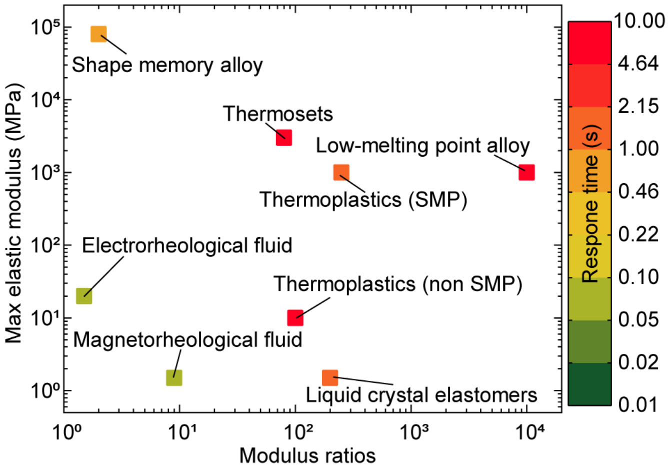

Figure S15. Stiffness properties of state-of-the-art variable stiffness materials. Relation among maximum elastic modulus, modulus ratio(maximum over minimum), and response time of reported and analyzed variable stiffness materials. Adapted from Wang et all (*9*).

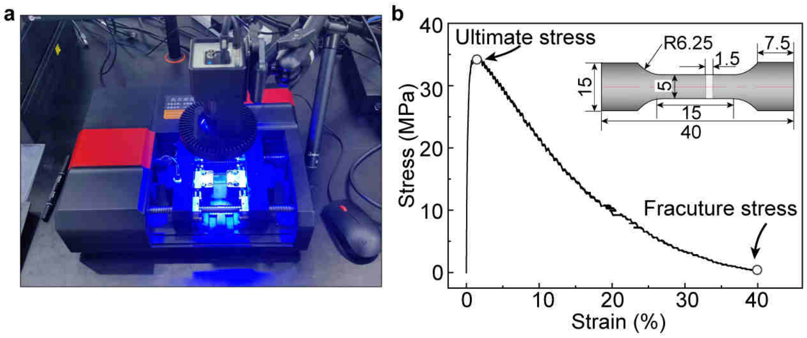

Figure S16. Mechanical properties testing of LMPA (Bolton 117). (**a**) Measurement system (IBTC-5000, CARE Measurement & Control Co., Ltd., China). (**b**)Stress-strain curve of LMPA

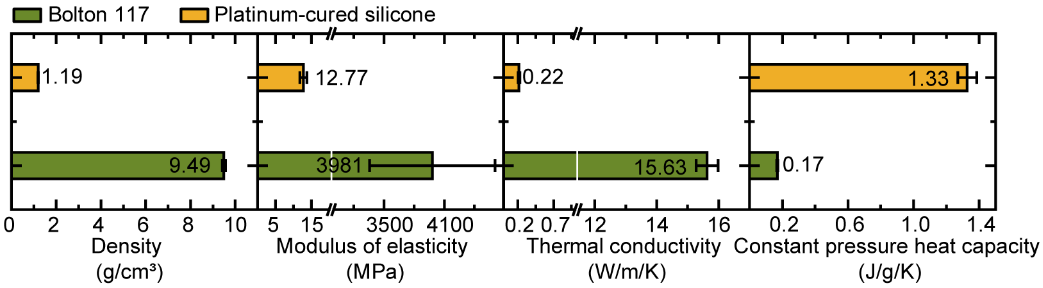

Figure S17. Physical properties of Platinum-cured silicone and LMPA (Bolton 117). The densities of both were measured by Archimedes' drainage method. The elastic modulus of Platinum-cured silicone was obtained using AFM, while that of material LMPA was determined by universal tensile testing machine. The thermal conductivity and constant pressure heat capacity of both materials were measured using a laser thermal conductivity meter.

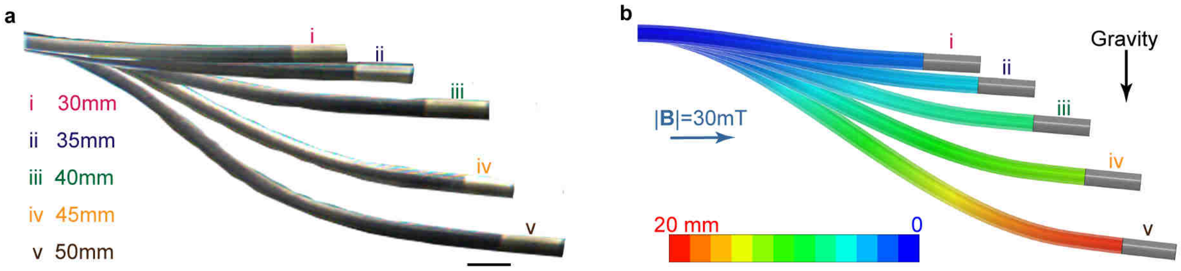

Figure S18. The influence of gravity on the flexible Guider's deflection induced by the applied magnetic field. The simulation results **(b)** are consistent with the experimental results **(a)**. Scale bars, 5 mm.

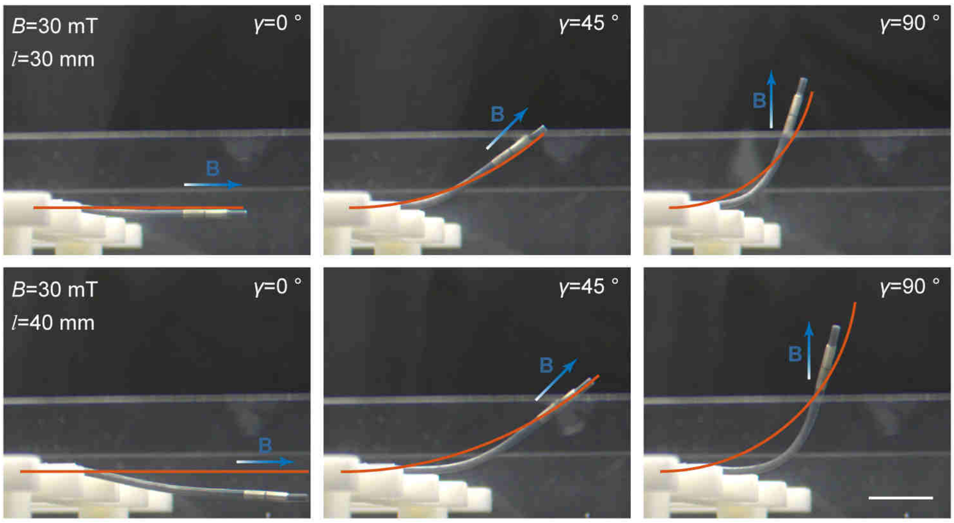

Figure S19. Deformation of the Guider under external magnetic fields. Comparison of real deformation and predicted curvature (orange) under different magnetic field inclination angles. Scale bars, 10 mm.

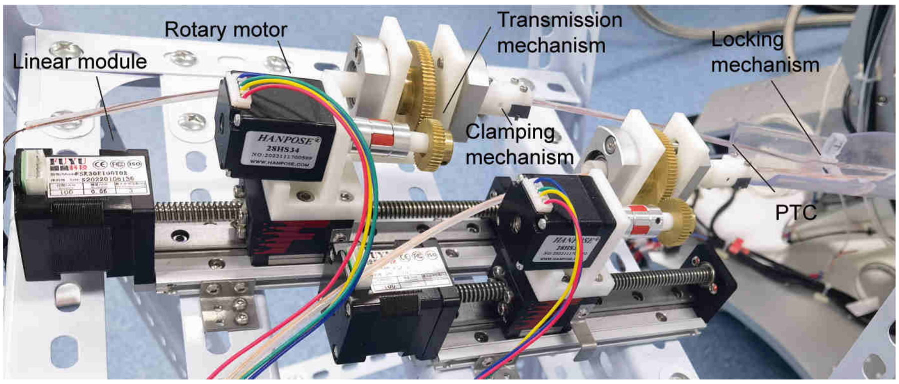

Figure S20. Structure and operational steps of the advancement unit. The advancement unit is composed of a linear module, a rotary motor, a transmission mechanism, a clamping mechanism, and a locking mechanism. The operational steps are as follows: i) The PTC is secured using the clamping mechanism, and then the linear module drives the PTC along the axial direction. ii) Upon reaching the limit position, the clamping mechanism is released and the locking mechanism is engaged. iii) With the PTC held in place by the locking mechanism, the linear module retracts to its initial position, ready for the next cycle of operation.

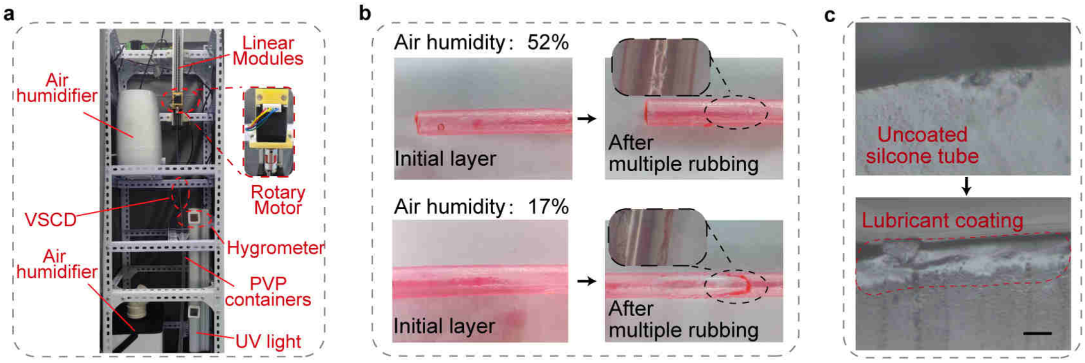

Figure S21. Coating and characterization of hydrogels for lubrication on PTC surface. (a) Coating equipment grows hydrogel on the PTC surface. **(b)** Air humidity ought to be within an appropriate range, otherwise, it will considerably reduce the bonding capability of the lubricated coating to the substrate. **(c)** PTC was wrapped in a lubricated layer under the microscope. Scale bars, 10 μm.

Supplementary Reference

1. A. Degani, H. Choset, A. Wolf, M. Zenati, Highly articulated robotic probe for minimally invasive surgery, in *2006 IEEE International Conference on Robotics and Automation (ICRA)* 4167-4172 (2006).
2. M. Cianchetti, Tommaso Ranzani, G. Gerboni, T. Nanayakkara, K. Althoefer, P. Dasgupta, A. Menciassi, Soft robotics technologies to address shortcomings in today's minimally invasive surgery: the STIFF-FLOP approach. *Soft Robot*. **1**, 122-131 (2014).
3. B. Yang, R. L. Baines, D. S. Shah, S. Patiballa, E. Thomas, M. Venkadesan, R. Kramer-Bottiglio, Reprogrammable soft actuation and shape-shifting via tensile jamming. *Sci. Adv.* **7**, eabh2073 (2021).
4. M. Mattmann, C. de Marco, F. Briatico, S. Tagliabue, A. Colusso, X. Chen, J. Lussi, C. Chautems, S. Pané, B. Nelson, Thermoset shape memory polymer variable stiffness 4D robotic catheters. *Adv. Sci.* **9**, 2103277 (2022).
5. M. Mattmann, Q. Boehler, X. Chen, S. Pané, B. Nelson, Shape memory polymer variable stiffness magnetic catheters with hybrid stiffness control, in *2022 IEEE/RSJ International Conference on Intelligent Robots and Systems (IROS)* 9589-9595 (2022).
6. J. Lussi, M. Mattmann, S. Sevim, F. Grigis, C. Marco, C. Chautems, S. Pané, J. Luis, Q. Boehler, B. Nelson, A submillimeter continuous variable stiffness catheter for compliance control. *Adv. Sci.*, **8**, 2101290 (2021).
7. Z. Xing, F. Wang, Y. Ji, D. McCoul, X. Wang, J. Zhao, A structure for fast stiffness-variation and omnidirectional-steering continuum manipulator. *IEEE Robot. Autom. Lett.* **6**, 755-762 (2020).
8. C. Chautems, A. Tonazzini, D. Floreano, B. Nelson, A variable stiffness catheter controlled with an external magnetic field, in *2017 IEEE/RSJ International Conference on Intelligent Robots and Systems (IROS)* 181-186 (2017).
9. L. Wang, Y. Yang, Y. Chen, C. Majidi, F. Iida, E. Askounis, Q. Pei, Controllable and reversible tuning of material rigidity for robot applications. *Mater. Today* **21**, 563-576 (2018).
