# Supplementary material for: Magnetic steering continuum robot for transluminal procedures with programmable shape and functionalities
Source: Nat Commun. 2024 May 4;15:3759. doi: 10.1038/s41467-024-48058-x (PMC11069526; doi:10.1038/s41467-024-48058-x)
Supplement: Supplementary file 17 — Reporting Summary [file 41467_2024_48058_MOESM17_ESM.pdf]

nature portfolio

Corresponding author(s): Hui Xie, Xianghe Meng, Hao Zhang

Last updated by author(s): Mar 29, 2024

Reporting Summary

Nature Portfolio wishes to improve the reproducibility of the work that we publish. This form provides structure for consistency and transparency in reporting. For further information on Nature Portfolio policies, see our Editorial Policies and the Editorial Policy Checklist.

Statistics

For all statistical analyses, confirm that the following items are present in the figure legend, table legend, main text, or Methods section.

n/a	Confirmed	
☐	☒	The exact sample size (n) for each experimental group/condition, given as a discrete number and unit of measurement
☐	☒	A statement on whether measurements were taken from distinct samples or whether the same sample was measured repeatedly
☒	☐	The statistical test(s) used AND whether they are one- or two-sided<br>*Only common tests should be described solely by name; describe more complex techniques in the Methods section.*
☒	☐	A description of all covariates tested
☐	☒	A description of any assumptions or corrections, such as tests of normality and adjustment for multiple comparisons
☐	☒	A full description of the statistical parameters including central tendency (e.g. means) or other basic estimates (e.g. regression coefficient) AND variation (e.g. standard deviation) or associated estimates of uncertainty (e.g. confidence intervals)
☒	☐	For null hypothesis testing, the test statistic (e.g. F, t, r) with confidence intervals, effect sizes, degrees of freedom and P value noted<br>*Give P values as exact values whenever suitable.*
☒	☐	For Bayesian analysis, information on the choice of priors and Markov chain Monte Carlo settings
☒	☐	For hierarchical and complex designs, identification of the appropriate level for tests and full reporting of outcomes
☒	☐	Estimates of effect sizes (e.g. Cohen's d, Pearson's r), indicating how they were calculated

Our web collection on statistics for biologists contains articles on many of the points above.

Software and code

Policy information about availability of computer code

Data collection	Videos were captured with the optical camera (acA2040-562 120um, Basler, Inc.), thermal imager (TPH21 Pro), ultrasonic imaging device (E2, SonoScape, Inc.) and X-ray imaging equipment (IPET007, Shenzhen Bestcare Biotechnology Co., Ltd.). Image processing and data collection were accomplished with LabVIEW (2020).
Data analysis	Abaqus 2017 and COMSOL 5.6 was used for simulation. Origin 2018 and Python 3.0 were used for analysis the data.

For manuscripts utilizing custom algorithms or software that are central to the research but not yet described in published literature, software must be made available to editors and reviewers. We strongly encourage code deposition in a community repository (e.g. GitHub). See the Nature Portfolio guidelines for submitting code & software for further information.

Data

Policy information about availability of data

All manuscripts must include a data availability statement. This statement should provide the following information, where applicable:

- Accession codes, unique identifiers, or web links for publicly available datasets
- A description of any restrictions on data availability
- For clinical datasets or third party data, please ensure that the statement adheres to our policy

The data generated in this study are provided in the main text, supplementary information, and source data file. Additional data are available from the corresponding author on request. Source data are provided with this paper.

Research involving human participants, their data, or biological material

Policy information about studies with human participants or human data. See also policy information about sex, gender (identity/presentation), and sexual orientation and race, ethnicity and racism.

Reporting on sex and gender	this information has not been collected
Reporting on race, ethnicity, or other socially relevant groupings	this information has not been collected
Population characteristics	not applicable
Recruitment	not applicable
Ethics oversight	not applicable

Note that full information on the approval of the study protocol must also be provided in the manuscript.

Field-specific reporting

Please select the one below that is the best fit for your research. If you are not sure, read the appropriate sections before making your selection.

☒ Life sciences ☐ Behavioural & social sciences ☐ Ecological, evolutionary & environmental sciences

For a reference copy of the document with all sections, see nature.com/documents/nr-reporting-summary-flat.pdf

Life sciences study design

All studies must disclose on these points even when the disclosure is negative.

Sample size	For cytotoxic tests, sample size was selected for a minimum of n=3.<br>The deployment and functionalization of the proposed continuum robot in vivo can only verify the effectiveness of this method. This is no sample size calculation.
Data exclusions	No data were excluded from the analyses.
Replication	All experiments were repeated a minimum of one time to confirm the repeatability of the findings. All repeats of experiments were successful.
Randomization	Not applicable. The application of the continuum robot in vivo can only verify the effectiveness of this method. There is no control group, and random method cannot be used.
Blinding	Not applicable. The application of the continuum robot in vivo can only verify the effectiveness of this method. There is no control group, and blind method cannot be used.

Reporting for specific materials, systems and methods

We require information from authors about some types of materials, experimental systems and methods used in many studies. Here, indicate whether each material, system or method listed is relevant to your study. If you are not sure if a list item applies to your research, read the appropriate section before selecting a response.

Materials & experimental systems

n/a	Involved in the study	
☒	☐	Antibodies
☐	☒	Eukaryotic cell lines
☒	☐	Palaeontology and archaeology
☐	☒	Animals and other organisms
☒	☐	Clinical data
☒	☐	Dual use research of concern
☒	☐	Plants

Methods

n/a	Involved in the study	
☒	☐	ChIP-seq
☒	☐	Flow cytometry
☒	☐	MRI-based neuroimaging

Eukaryotic cell lines

Policy information about cell lines and Sex and Gender in Research

Cell line source(s)	Human umbilical vein endothelial HUVEC cells (PCS-100-013), American Type Culture Collection.
Authentication	All cell lines were authenticated and characterized by the supplier
Mycoplasma contamination	No Mycoplasma contamination.
Commonly misidentified lines (See ICLAC register)	None

Animals and other research organisms

Policy information about studies involving animals; ARRIVE guidelines recommended for reporting animal research, and Sex and Gender in Research

Laboratory animals	We performed experiments in a 15 kg male Large White pig.(Experimental Animal Center of Harbin Veterinary Research Institute)
Wild animals	No wild animals were used in this study.
Reporting on sex	This information has not been collected
Field-collected samples	No field-collected samples were used in this study.
Ethics oversight	All animals were used in accordance with the Guide for Care and Use of Laboratory Animals. Animal experiments were permitted by the Experimental Animal Welfare Ethics Committee of Harbin Institute of Technology (no.: IACUC-2023058)

Note that full information on the approval of the study protocol must also be provided in the manuscript.

Plants

Seed stocks	Not applicable.
Novel plant genotypes	Not applicable.
Authentication	Not applicable.
